# Salinity Regulation of the Interaction of Halovirus SNJ1 with Its Host and Alteration of the Halovirus Replication Strategy to Adapt to the Variable Ecosystem

**DOI:** 10.1371/journal.pone.0123874

**Published:** 2015-04-08

**Authors:** Yunjun Mei, Congcong He, Yongchi Huang, Ying Liu, Ziqian Zhang, Xiangdong Chen, Ping Shen

**Affiliations:** 1 School of Chemical and Environmental Engineering, Wuhan Polytechnic University, Wuhan, Hubei, China; 2 School of Biology and Pharmaceutical Engineering, Wuhan Polytechnic University, Wuhan, Hubei, China; 3 State Key Laboratory of Virology, College of Life Science, Wuhan University, Wuhan, Hubei, China; The Chinese University of Hong Kong, HONG KONG

## Abstract

Halovirus is a major force that affects the evolution of extreme halophiles and the biogeochemistry of hypersaline environments. However, until now, the systematic studies on the halovirus ecology and the effects of salt concentration on virus-host systems are lacking. To provide more valuable information for understanding ecological strategies of a virus-host system in the hypersaline ecosystem, we studied the interaction between halovirus SNJ1 and its host *Natrinema* sp.J7-2 under various NaCl concentrations. We found that the adsorption rate and lytic rate increased with salt concentration, demonstrating that a higher salt concentration promoted viral adsorption and proliferation. Contrary to the lytic rate, the lysogenic rate decreased as the salt concentration increased. Our results also demonstrated that cells incubated at a high salt concentration prior to infection increased the ability of the virus to adsorb and lyse its host cells; therefore, the physiological status of host cells also affected the virus-host interaction. In conclusion, SNJ1 acted as a predator, lysing host cells and releasing progeny viruses in hypersaline environments; in low salt environments, viruses lysogenized host cells to escape the damage from low salinity.

## Introduction

Viruses are the most abundant biological entities in aquatic systems; the estimated number of virus-like particles in the biosphere is more than 10^31^, and viruses outnumber cellular organisms by at least one order of magnitude [1, 2]. Archaeal viruses were first discovered in 1974 [3], the number of studied archaeal viruses is now approximately 100 [4–6], accounting for only approximately 1–2% of the known prokaryotic and eukaryotic viruses. Thus, the focus on archaeal viruses is limited, and there are many questions in our understanding of archaeal viruses and their ecological functions [5, 7–9].

Haloviruses inhabit hypersaline environments, such as salt lakes and salterns, and can include saturated salt solutions. The number of virus-like particles in hypersaline environments is approximately 10^7^–10^9^ particles per ml, and they are the dominant predator in such ecosystems [10, 11]. Haloviruses possess a wide diversity in morphology [6, 12–14] and genomic structure [9, 15–20]. Although the pioneering work began several decades ago [21, 22], studies that address viral ecology and the investigation of the effects of salt concentration on virus-host systems are still rare [8, 19]. Exploring and elucidating the interaction between haloviruses and their hosts is only in the preliminary phase, and some of major areas have been ignored, particularly concerning the initial stages of virus infection [8].

*Natrinema* sp. J7-2 was isolated from a salt mine in China [23], where the species of genera *Natrinema*, *Haloterrigena* and *Halorubrum* are predominant haloarchaeal organisms [24]. Halovirus SNJ1 (circular double-strand DNA, 16.4 kb) is a spherical (S1 Fig), temperate membrane-containing archaeal virus only infecting *Natrinema* sp. J7-2, which is a provirus existing early in *Natrinema* sp. J7-1 as a plasmid [25–27]. Halovirus SNJ1 belongs to an SH1-like virus lineage based on the two major capsid proteins folds and a similar genome and packaging of ATPase. The members of this lineage include *Haloarcula hispanica* virus SH1 [28], *Thermus thermophilus* phage P23-77 [29], *Thermus aquaticus* phage ϕIN93 [30], *Salisaeta icosahedral* phage1 and several proviruses [29, 31].

Based on our previous research, in this study, we report the interaction between SNJ1 and its host under varying salinity, which is a crucial environmental factor in hypersaline ecosystems. Our results will provide important information regarding the dynamic changes of the virus-host system in extremely hypersaline environments and provide insight into the ecology of extremely halophilic environments.

## Materials and Methods

### Virus, cell, media, and growth conditions

The haloarchaeal strain and virus used in this study were *Natrinema* sp. J7-2 and halovirus SNJ1, respectively [26, 27]. Cells were grown aerobically at 37°C in different media. All media contained the same components, with an exception of the NaCl concentration (per liter): 30 g of MgCl_2_.6H_2_O, 80 ml of 1 M Tris-HCl (pH 7.2), 2.5 g of lactalbumin hydrolysate (Difco laboratories, Detroit, USA), and 2 g of Bacto yeast extract (Difco laboratories, Detroit, USA). Media contained varying amounts of NaCl (per liter) 120 g, 150 g, 180 g, 200 g, 230 g, 250 g, 270 g, or 300 g were designated as % of corresponding medium, e.g., 12%,15%, and 18% NaCl. Bacto agar (Difco laboratories, Detroit, USA) was used to prepare plates (12 g/l) or top-layer agar (4.5 g/l). SNJ1 was stored at 4°C.

### The effect of salinity to SNJ1 and its host

The determination of the effect of salinity to the interaction of SNJ1 with its host can be executed only in conditions that do not have adverse effects either to the virus or to the host. To acquire this parameter, the remaining infective activity of SNJ1 in different salinity solutions and the growth rate of *Natrinema* sp. J7-2 in varying salinity media were detected. SNJ1 was incubated in solutions with variable NaCl concentrations at 20°C, the samples were extracted from the buffer solutions at the indicative time. The viral titers were determined by plaque assay as follows: 100 μl of early logarithmic phase cultures of *Natrinema* sp. J7-2 incubated in 30% NaCl were infected with the respective properly diluted samples. The adsorption mixture was kept at 37°C without agitation, with NaCl concentration of 30%. At 1-h p.i., the mixture were added to 4 ml pre-heated top-layer agar containing 25% NaCl and poured onto the pre-prepared bottom-layer agar (containing 25% NaCl and 12 g/l agar). The plates were incubated at 37°C for 2 days. Plaque formation was checked subsequently. *Natrinema* sp. J7-2 were grown aerobically at 37°C in different media, sampled at the indicative time, and then assayed via the optical density at 550 nm. All experiments were performed in triplicate.

### Adsorption efficiency determination

To determine the adsorption efficiency, early logarithmic phase cultures of *Natrinema* sp. J7-2 (*Natrinema* sp. J7-2 approximately 2.5×10^8^, 2.0×10^8^, 1.7×10^8^ colony-forming units (CFU) ml^-1^ incubated in 18% NaCl, 25% NaCl, and 30% NaCl, respectively) were infected with a multiplicity of infection (MOI) of 10^-4^ (under this condition, one host cell is adsorbed by one virus at most, so the number of plaques is able to reflect the adsorbed viruses). The adsorption mixture was kept at 37°C without agitation, with the NaCl concentration in the range from 18% to 30%. At 1-h post infection (p.i.), the mixture was centrifuged (13,523 × *g* at 4°C for 6 min; Eppendorf 5424R, Hamburg, Germany), and then the supernatant was removed. The pellet was washed twice, resuspended, and then poured into the top-layer agar for a plaque assay (the salinity of the top-layer agar is 25% NaCl). All adsorption efficiency experiments were performed in triplicate.

### Thin-section electron microscopy

The protocol of cell fixation and embedding followed previous method [32], with modifications. 2 ml of early logarithmic phase cultures of *Natrinema* sp. J7-2 incubated in 18%, 25% and 30% NaCl (cells cultured in 18% NaCl for 22 h, in 25% NaCl for 26 h, and in 30% for 28 h, respectively) was centrifuged (13,523 × *g* at 4°C for 6 min; Eppendorf 5424R, Hamburg, Germany), and then the supernatant was removed. The pellets were covered with a 4% solution of formaldehyde that contained the identical NaCl concentration to that of the respective culture media at pH 7.0, stored at 4°C for 8 h. Next, the pellets were washed 4 times at 30 min intervals with the corresponding NaCl concentration solution and then exposed to 1% osmium tetroxide with corresponding NaCl concentration for 2 h, at pH 7.0. The specimens were then washed in the corresponding NaCl solution and treated with 2% aqueous solution of uranyl nitrate for 30 min. After several washings with water, the fixed specimens were dehydrated with gradient ethanol solutions and embedded in Epon for sectioning. Thin sections were cut with a diamond knife and then mounted on Formvar films that were coated with carbon. The sections were double-stained with magnesium uranyl acetate and lead citrate. The micrographs were obtained using a HITACHI-HT 7700 (Hitachi High-Tech, Tokyo, Japan) transmission electron microscope operating at 80 kV.

### Single-step growth curve of SNJ1 incubated in media with varied salinity

Samples of 1 ml of early logarithmic phase cultures of *Natrinema* sp. J7-2 incubated in 18%, 25%, and 30% NaCl liquid media were infected with SNJ1 with an MOI of 50 at 37°C; the NaCl concentration of adsorption was 30% (under these conditions, the percentage of infected cells over 95%). At 1-h p.i., the infected cells were collected through centrifugation (13,523 × *g* at 4°C for 6 min; Eppendorf 5424R, Hamburg, Germany), washed twice, and then the infected cells were re-inoculated into their respective fresh pre-heated liquid media at 37°C with low aeration. The samples were taken from the cultures of the liquid media at indicative intervals for viral assay. The samples were divided into two aliquots: one for detecting the extracellular virus and infective centers, and the other for assaying extracellular and mature intracellular virus particles. In the latter case, the cells were broken ultrasonically (Φ3, 200 W, working time 3 s and interval 3 s, total 2 min, at or below10°C; Scientz-IID, Ningbo, China).Viral titers were determined by the plaque assay as described above. All experiments were performed in triplicate.

### Regulation of the replication cycles of SNJ1 by salinity

To investigate the correlation between the replication cycles and the salinity, sensitive cells were infected with an MOI of 50, according to the procedures of the single-step growth curve. At 1-h p.i., cells were collected using a centrifuge (13,523 × *g*, at 4°C for 6 min; Eppendorf 5424R, Hamburg, Germany), washed twice, and then resuspended. Each type of infected cell was spread over plates with 18%, 25%, and 30% NaCl for counting colonies. All plates were kept at 37°C for 7 days, and then the colonies were counted and the lytic rate was calculated. One-hundred colonies were randomly chosen for each treated sample and the given primers were used to measure lysogen and to subsequently analyze the lysogenic rate.

For example, cells incubated in the 18% NaCl case were infected according to the above description. The infected cells were centrifuged, washed, resuspended and spread over 18%, 25%, and 30% NaCl solid media for colony counts, using the same procedure for the cells incubated in the 25% and 30% NaCl cases. Uninfected cells that were spread over 25% NaCl solid medium served as a control.

### Imitation of the interaction of SNJ1 and its host in natural niches

Salinity in natural niches is often affected by rainfall or evaporation; thus, the salinity of natural niches fluctuates periodically. To investigate the dynamics of the virus-host system with the alteration of salinity, three NaCl concentrations (18%, 25%, and 30%) were chosen, and then the lytic and lysogenic rates were analyzed using infected host cells with an MOI of 10. The salinity levels for host growth, SNJ1 adsorption and colony formation were identical.

### Analysis of the lytic rate and lysogenic rate

The lytic rate and lysogenic rate were determined using the following equations:

$$total\;lytic\;rate\;\left(A\right):A=\frac{\left(a-b\right)}{a}\times 100\%$$ 1

$$total\;lysogenic\;rate\;\left(B\right):B=\frac{c}{100}\times \frac{b}{a}\times 100\%$$ 2

total lysogen (C):C=B×a 3

Where a is the total CFU of 1 ml of the control, b is the number of viable cells derived from 1 ml of the infected cells, and c is the number of lysogens randomly picked out from 100 viable cells. The lytic rate and lysogenic rate were based on triplicate measurements.

## Results

### The effect of salinity on SNJ1 and its host

**Fig 1:**
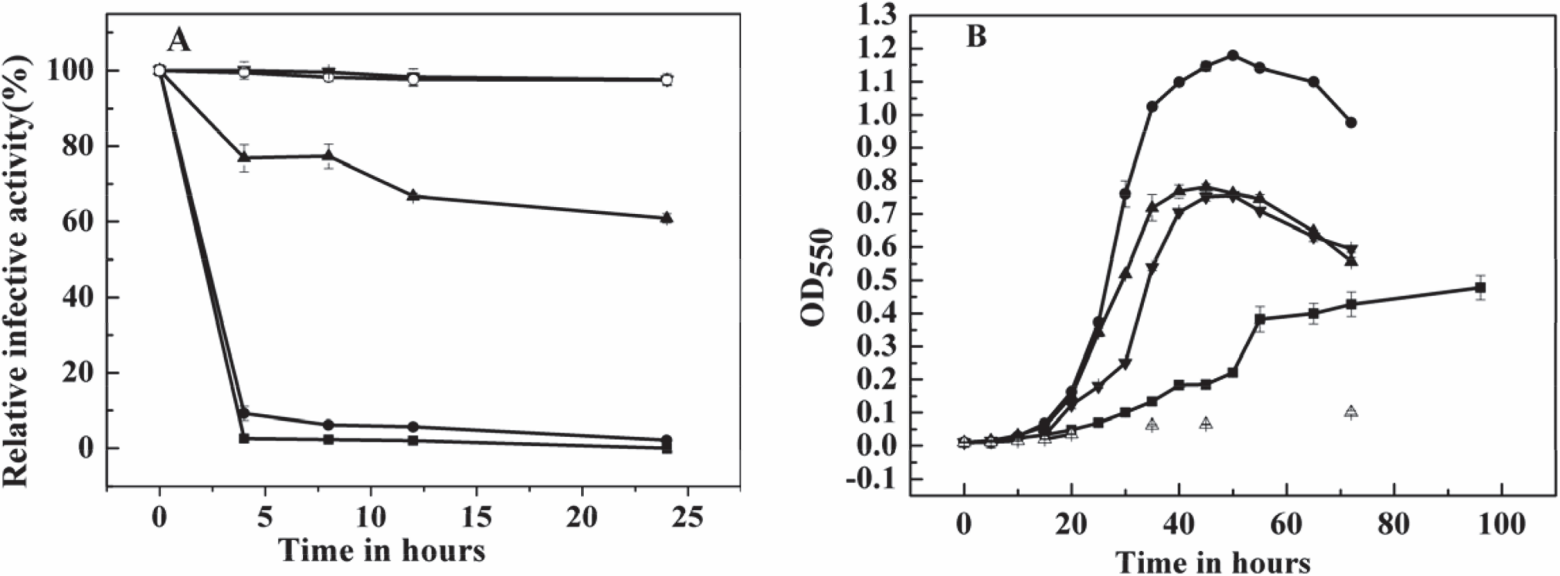
The effect of salinity on the infective activity of halovirus SNJ1 and the growth of its host cells. (A) the remaining infective activity of halovirus SNJ1 under different NaCl concentrations at indicative time; ■,●,▲,▼, and ○ represent the remaining infective activity in 12%, 15%, 18%, 25%, and 30% NaCl, respectively. (B) the growth curves of *Natrinema* sp. J7-2 under different NaCl concentrations at indicative time; the symbols △, ■, ●, ▲, and ▼ denote the growth of the host cells in 12%, 15%, 18%, 25%, and 30% NaCl, respectively.

The haloarchaeal strains and haloviruses flourish in hypersaline environments, and the salinity of the ecosystem affects their viability. To determine the effect of salinity on SNJ1, the remaining infective activities were executed. The infective activity decreased drastically within 24 h when the salinity was lower than 18%: 60.8% and 2.2% in 18% and 15% NaCl solutions after 24 h, respectively. In 12% NaCl solution, the infective activity was nearly zero (Fig 1A). Similar assays were used to determine the viabilities of the host cells within the same range of NaCl concentration. The growth of *Natrinema* sp J7-2 exhibited different growth rates in media containing variable salinities, and the host cells grew well in all media, except in 12% NaCl (Fig 1B). The highest growth rate was in 18% NaCl, followed by those in 25%, 30% and 15% NaCl. However, in 12% NaCl, the optical density at 550 nm, which was slightly increased after 72 h, was lower than 0.1. Interestingly, the optical density of cells in 12% medium increased to 0.5–0.6 after 11 days, which suggested that partial host cells were still alive, even when kept in 12% NaCl. Comparing the results of halovirus infective activities with host growth rates in various salinities, we found that the host cells were more tolerant to an altered NaCl concentration than SNJ1.

### Adsorption efficiency at different salt concentrations

**Fig 2:**
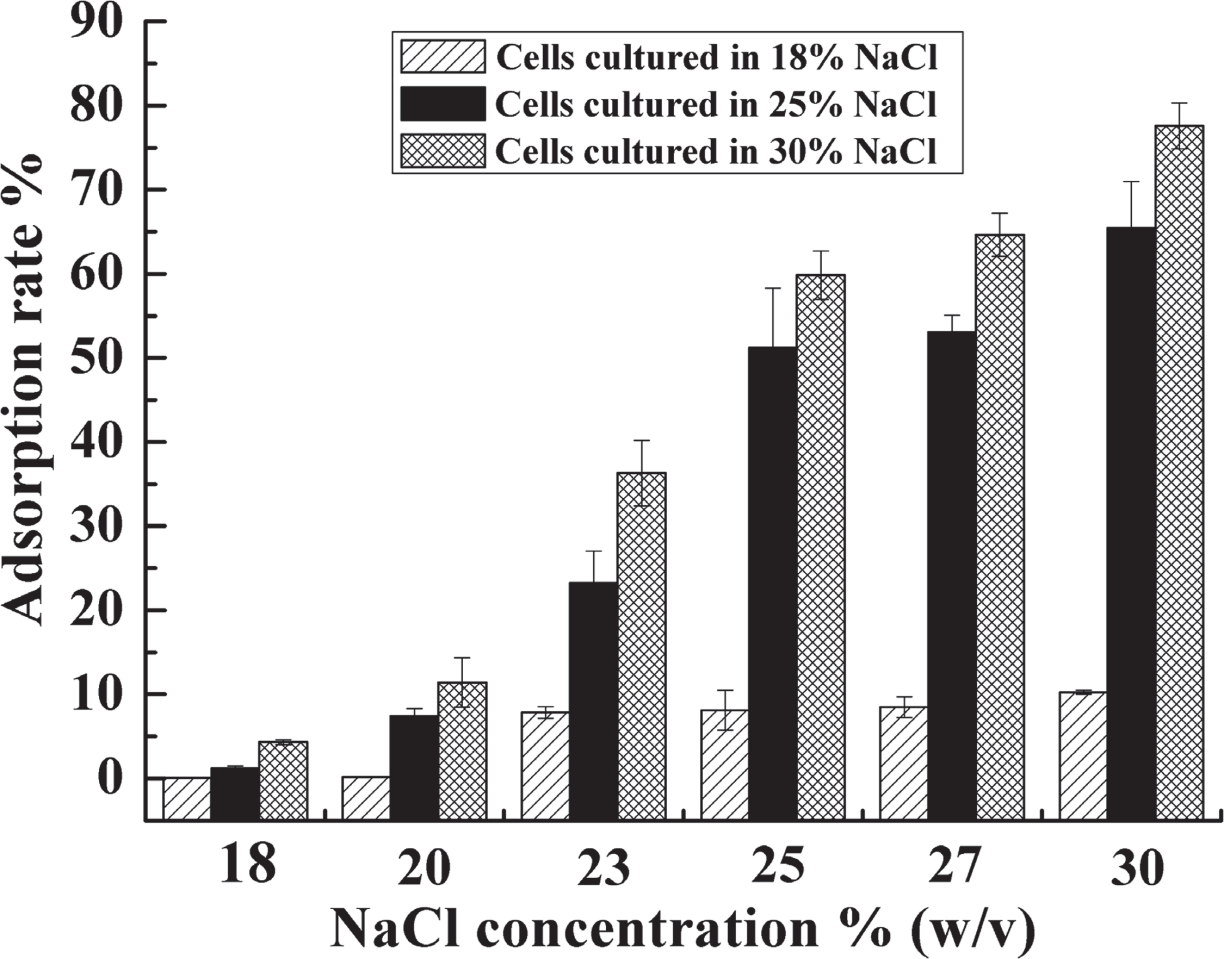
Adsorption efficiency of SNJ1 under different salt concentrations.

**Fig 3:**
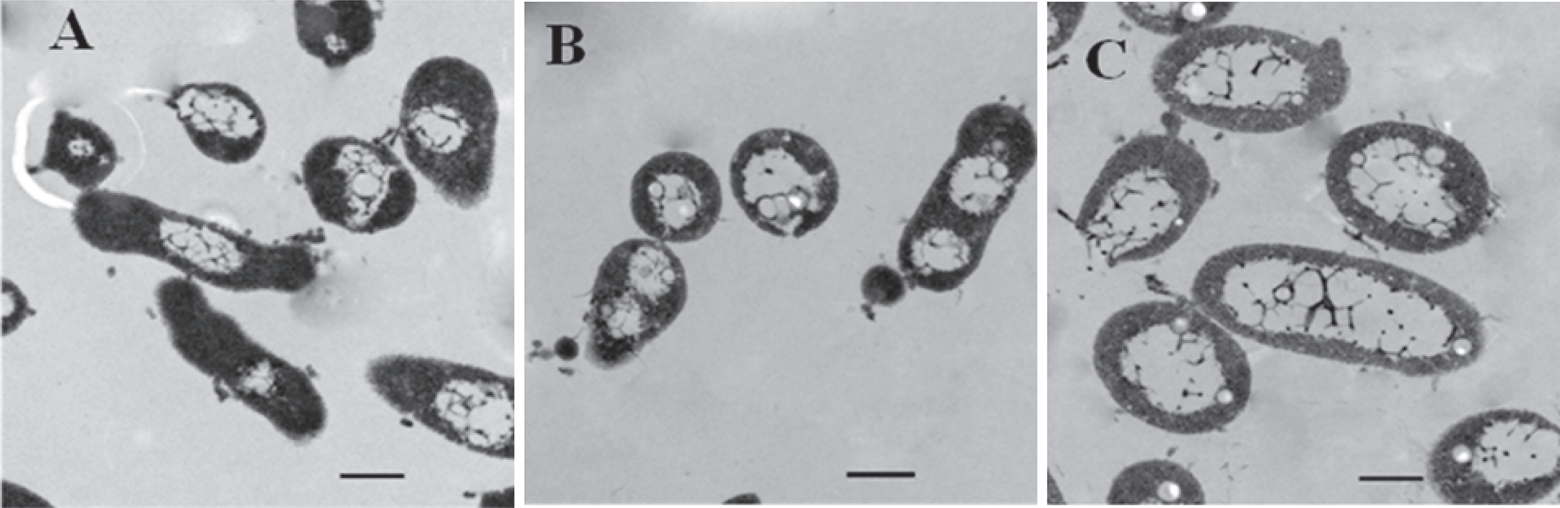
Thin section electron micrographs of *Natrinema* sp. J7-2 for cells incubated in media with different salinity. (A) cells incubated in 18% medium; (B) cells incubated in 25% medium; (C) cells incubated in 30% medium. The scale bars represent 500 nm.

For all host cells, regardless of being incubated in 18% NaCl, 25% NaCl, or 30% NaCl prior to be infected, the adsorption efficiency was affected by salinity and increased with salt concentration. This result showed that the ionic strength of the adsorption process altered the adsorption efficiency. When the salinity of adsorption was identical, the adsorption efficiency of cells incubated in 30% NaCl was greater than those incubated in 25% and 18% NaCl (Fig 2), which indicated that the physiological characteristics of cells prior to being infected also affected the adsorption rate. Specifically, Fig 2 shows that there was a large variation in the adsorption rate, ranging from 0.1% to 77.6%. Cells incubated in 30% medium were most susceptible to SNJ1, with the adsorption rates ranging from 11.3% to 77.6%. Cells inoculated in 18% medium were the least susceptible, with adsorption efficiency ranging from 0.1% to 10.3%. Fig 3 displays the thin section electronic micrographs of *Natrinema* sp. J7-2 incubated in 18%, 25%, and 30% NaCl. All the cells developed central light areas, which occupied a an increasing fraction of the total cell volume with increasing salinity. Conversely, the cytoplasm gradually decreased with increasing salinity, and the composite structure of cell wall and the periplasmic space also attenuated (Fig 3A, 3B and 3C).These findings illustrate that NaCl acts through two pathways: one is changing the ionic strength of adsorption, and the other is altering the physiological status of the host cells.

### Single-step growth curves of SNJ1 at various salinities

**Fig 4:**
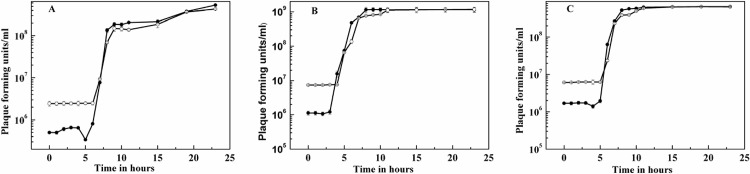
Single-step growth curves of halovirus SNJ1 incubated in media with differing salinity. (A) cells incubated in 18% medium; (B) cells incubated in 25% medium; (C) cells incubated in 30% medium. ●, extracellular and mature intracellular phage; ○, extracellular phage and infective centers.

In the single-cycle growth experiments using an MOI of 50, the number of virus progeny and the latent period varied when the infected *Natrinema* sp. J7-2 cells were incubated in media containing different NaCl concentrations (Fig 4). In the 18% NaCl case, the eclipse period was 5 h, and the latent period was 6 h (Fig 4A). In the 25% NaCl case, the eclipse period was 3 h, and the latent period was 4 h (Fig 4B). In the 30% NaCl case, the eclipse period was 4 h, and the latent period was 5 h (Fig 4C). These results indicated that the eclipse period and latent period of the infected cells incubated in the 25% NaCl medium were the shortest, followed by infected cells incubated in the 30% NaCl medium, and then the infected cells in the 18% NaCl medium. Furthermore, there was a difference in the burst size. In the 25% NaCl medium, the burst size was the largest, with 100–150 viruses per cell. Those cells incubated in the 30% NaCl solution had 40–70 viruses per cell, while there were 20–50 viruses per cell in the 18% NaCl medium. Earlier, we found SNJ1 was able to form visible plaques with a diameter approximately 2 mm at 48-h p.i. in soft agar with the 25% NaCl medium, while in the top agar with the 18% and 30% NaCl media, the plaques were smaller and delayed until 72-h p.i. The single-step growth curves indicated that these phenomena are ascribed to the differences in the burst size and the latent period.

### Regulation of the replication cycles of SNJ1 by salinity

Salinity not only affected the adsorption rate of virus SNJ1 and the physiological characteristics of the host but also affected the replication cycles of SNJ1 when it entered the host cells. The results are presented in Table 1.

**Table 1:** Regulation of the replication cycles of halovirus SNJ1by salinity (w/v, %).

Salinity of liquid	CFU/ml(×10^8^)	Salinity of solid plate	CFU of viable cell /ml	Total lytic rate(A)%	Total lysogenic rate(B)%	Total lysogen(B×a)
18	2.5±0.2	18	(1.5±0.1)×10^8^	41.3±5.1	7.9±0.1	(2.0±0.5)×10^7^
18	2.5±0.2	25	(7.1±0.8)×10^6^	97.2±0.3	0.5±0	(1.3±0)×10^6^
18	2.5±0.2	30	(3.6±0.6)×10^6^	98.5±0.2	0.3±0	(8.3±0.5)×10^5^
25	2.0±0.2	18	(3.4±0.2)×10^7^	82.9±0.9	5.2±0.5	(1.0±0.9)×10^7^
25	2.0±0.2	25	(4.4±0.5)×10^6^	97.8±0.3	0.4±0	(8.7±0.5)×10^5^
25	2.0±0.2	30	(1.3±0.2)×10^5^	99.9±0	0.03±0	(7.5±0.2)×10^4^
30	1.7 ±0.1	18	(3.1±0.4)×10^6^	98.2±0.2	0.62±0	(1.1±0.2)×10^6^
30	1.7 ±0.1	25	(1.9±0.2)×10^6^	99.9±0	0.2±0	(3.4±0.3)×10^5^
30	1.7 ±0.1	30	(1.7±0.2)×10^4^	100±0	0.005±0	(8.5±0.2)×10^3^

In the case of cells incubated in the 18% NaCl prior to being infected, the cells were infected with halovirus SNJ1 and spread over 18%, 25%, and 30% NaCl solid media. The lytic rate of cells increased with increasing NaCl concentration in solid media, ranging from 41.3% to 98.5%, but the lysogenic rate exhibited the opposite results: the lysogenic rate decreased with increasing NaCl concentration of the solid media, from 7.9% to 0.3%. The variations of lytic rate and lysogenic rate were attributed to the salinity gradient of the solid media because the treatment was identical except for the salinity of the solid media.This result illustrated the salinity regulation of the replication cycles of SNJ1. This pattern was similar to those cells incubated in the 25% and 30% NaCl media, and the lytic rate was even as high as 99.9%, and the lysogenic rate was as low as 0.005%.

All the results support the conclusion that the replication cycles of SNJ1 were regulated strictly by the salinity when it entered into the host cells. That is, low salinity was beneficial for the virus to lysogenize, and higher salinity was favorable for the virus to lyse.

### Imitation of the interactions of SNJ1 and its host in natural niches.

Lytic and lysogenic rates for cells infected by SNJ1at non-varying NaCl concentrations are presented in Table 2.

**Table 2:** Imitation of the interactions of SNJ1 and its host in natural niches.

Salinity of liquid media, adsorption and solid plate (w/v, %)	CFU/ml(×10^8^)	CFU of viable cell /ml	Total lytic rate(A)%	Lysogenic rate(B) %	Lysogen(B×a)
18	2.5±0.210^8^	(1.6±0.1)×10^8^	36.3±0.4	5.4±0.3	(1.4±0.7)×10^7^
25	2.0±0.2	(6.3±1.5)×10^6^	96.9±0.8	0.2±0	(4.8±0.4)×10^5^
30	1.7 ±0.1	(1.0±0.1)×10^5^	99.9±0	0.005±0	(8.5±0.9)×10^3^

In the low salinity treatment (18%), the lytic rate remained at a lower level, the lysogenic rate maintained a relatively higher level, and the vast majority of host cells were uninfected. In such a situation, lysogeny was favorable for the halovirus SNJ1 because it did not tolerate the low salinity (below 15% or even lower), while its host which grew well in the 15% NaCl medium. Of course, 18% salinity level also provided a refuge for the host cells due to the low lytic rate. However, for the cells in the 25% and 30% NaCl media, SNJ1 played a role as a predator because almost all the infected cells were broken. These findings also indicated that high salinity was favorable for SNJ1 to lyse its host cell, but low salt was beneficial to enter the lysogenic pathway.

## Discussion

The halovirus-haloarchaea interaction predominates in the hypersaline ecosytem, indicating that the interactions between haloviruses and their hosts are yet to be well understood, particularly concerning the initial stages of virus infection [8]. Here, we focus on the viability of the virus and the host, the adsorption rate, the single-step growth curve, and the replication cycles of SNJ1 under different NaCl concentrations.

We found that SNJ1 was less tolerant to altered NaCl concentration than its host cells (Fig 1), opposite to the behavior of previously reported haloviruses, for example, HCTV-1, HHTV-1, HHPV-1, HRTV-1, SCTP-1, SCTP-2 and SH1 [34], Hh-1an Hh-3 [35], and Ja.1[33]. Considering SNJ1 is a membrane-containing archaeal virus [27], the viral infective activity rapidly decreases because the membrane of the virus is broken due to the low ionic strength. However, the host cells have a reasonable regulation mechanism to adapt the alteration of the ionic strength [36–38]. As a result, it is very interesting to explore how SNJ1adapts to variations in the salinity, and how the degree the salinity affects the interaction between the halovirus SNJ1 and its host cells.

The adsorption rate of SNJ1 increased with increasing salinity (Fig 2), which was similar to the behavior of halovirus SCTP-1 [34], but differed from the behavior of other reported haloviruses. In the case of halovirus Hs1, the adsorption rate rapidly decreased with increasing salinity [21], and haloviruses HHTV-, HCTV-1, HHPV-1 and SCTP -2 displayed multiple adsorption responses to altered salt conditions and a maximal host cell binding at a particular NaCl concentration [34]. Other reports indicated that the adsorption rate was not affected significantly by changes in the ionic strength [22, 35]. Interestingly, the cells incubated in high salinity prior to being infected were more susceptible than the cells incubated in low salinity, with the other treatments being identical. The thin section electron micrographs indicated that the cellular structure of the host cells incubated in different media was quite different (Fig 3). Consequently, we infer that the physiological and biochemical levels of the host, such as the cell envelope (S-layer [39–41]), play a key role in SNJ1-binding to the host cells. Recently, numerous and varied examples of post-translational modifications were reported in the domain Archaea [42–48]. Tests of *Haloferax volcanii*, as a model system, demonstrated that the S-layer post-translational modification is a functionally adaptive response to the environmental salinity [49–51]. Perhaps divergences exist in the composition and decoration in the S-layer of *Natrinema* sp.J7-2 when it is cultured in the different salinity media, which affects the viral absorption to the host cells.

In addition, the salinity also affected the viral life cycle. SNJ1 entered into the lytic cycle in the high-salt virus-host system, the burst size increased, and the latent period shortened; while the lysogenized host cells in the low-salt virus-host system, the burst size was small and the latent phase was long (Fig 4 and Tables 1 and 2). This survival strategy of SNJ1 may be an evolved result to adapt to the variable salinity in the natural environment. When the natural niches are at a high salinity level during a drought, the host cells reach high population densities, and SNJ1 has the opportunity to rapidly propagate to enlarge the population density through shortening the eclipse and latent period, acting as a predator. While the salinity is reduced after a rainfall, SNJ1 become benign via prolonging period of lytic cycle and lysogeny to reduce the harm to its host cells. Viral DNA is preserved maximally and escapes from extinction because the host cell is able to tolerate lower salinity stress than halovirus SNJ1 (Fig 1). The observations of SNJ1 were different from those of other haloviruses [21, 22, 33, 35, 52] because they have different responses to the alteration of salinity. All of the results suggest SNJ1 has evolved to adapt to the constantly changing ionic strengthen in natural niches: in the high-salt virus-host system, SNJ1 exhibits a high adsorption rate and lyses its host cells, whereas in the low-salt virus-host system, SNJ1 mainly lysogenizes its host cells. These findings provide novel information regarding the haloviral survival strategy.

## Supporting Information

S1 Fig (TIF)
