# Supplementary figures and images for: Salinity Regulation of the Interaction of Halovirus SNJ1 with Its Host and Alteration of the Halovirus Replication Strategy to Adapt to the Variable Ecosystem
Source: PLoS One. 2015 Apr 8;10(4):e0123874. doi: 10.1371/journal.pone.0123874 (PMC4390326; doi:10.1371/journal.pone.0123874)

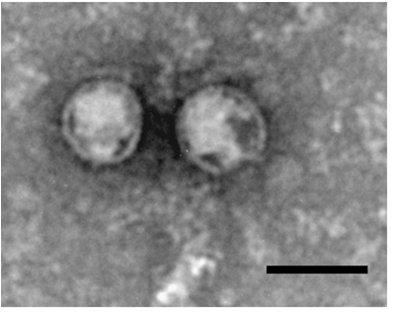

Supplement: S1 Fig — (TIF) [file pone.0123874.s001.tif]
